# Exploring the Relationship between Plasma Adiponectin, Gender, and Underlying Diseases in Severe Illness

**DOI:** 10.3390/biomedicines11123287

**Published:** 2023-12-12

**Authors:** Patricia Mester, Ulrich Räth, Stephan Schmid, Martina Müller, Christa Buechler, Vlad Pavel

**Affiliations:** Department of Internal Medicine I, Gastroenterology, Hepatology, Endocrinology, Rheumatology, and Infectious Diseases, University Hospital Regensburg, 93053 Regensburg, Germany; patricia.mester@klinik.uni-regensburg.de (P.M.); ulrich.raeth@stud.uni-regensburg.de (U.R.); stephan.schmid@klinik.uni-regensburg.de (S.S.); martina.mueller-schilling@klinik.uni-regensburg.de (M.M.); vlad.pavel@klinik.uni-regensburg.de (V.P.)

**Keywords:** COVID-19, adiponectin, liver cirrhosis, sex, pancreatitis, survival

## Abstract

Adiponectin is low in obesity, plays a crucial role in metabolic health, and, moreover, possesses immunoregulatory properties. However, studies examining its levels in patients with systemic inflammatory response syndrome (SIRS) or sepsis have yielded conflicting results. While females typically have higher systemic adiponectin levels than males, research on sex-specific associations in this context is limited. In this study of 156 SIRS/sepsis patients, including those with liver cirrhosis, we aimed to explore the relationship between plasma adiponectin, body mass index (BMI), gender, disease severity, and underlying etiological conditions. Our findings revealed that patients with liver cirrhosis, who are susceptible to infections, exhibited elevated circulating adiponectin levels, irrespective of sex. When excluding cirrhosis patients, plasma adiponectin levels were similar between male SIRS/sepsis patients and controls but lower in female patients compared to female controls. Plasma adiponectin was inversely related to BMI in female but not male patients. Further analysis within the non-cirrhosis subgroup demonstrated no significant differences in adiponectin levels between sexes among SIRS, sepsis, and septic shock patients. Ventilation, dialysis, and vasopressor therapy had no discernible impact on adiponectin levels in either sex. A negative correlation between adiponectin and C-reactive protein (CRP) existed in males only. Notably, patients with pancreatitis showed the lowest plasma adiponectin concentrations, although sex-specific differences were not significant. Infection with Gram-negative or Gram-positive bacteria had minimal effects on plasma adiponectin levels in both sexes. However, infection with the severe acute respiratory syndrome coronavirus type 2 led to decreased adiponectin levels in females exclusively. Multivariate analysis considering all factors affecting plasma adiponectin levels in males or females identified BMI in females and CRP levels in males to predict plasma adiponectin levels in SIRS/sepsis patients. Additionally, our study observed a trend where the 25 patients who did not survive had higher plasma adiponectin levels, particularly among males. In summary, our investigation highlights the influence of underlying diseases and sex on plasma adiponectin levels in SIRS/sepsis patients, shedding light on potential implications for disease management and prognosis.

## 1. Introduction

Sepsis is a severe condition characterized by elevated systemic levels of both pro- and anti-inflammatory cytokines [1,2].

Severe acute respiratory syndrome coronavirus type 2 (SARS-CoV-2) infection is a more recent cause of sepsis, and patients often develop pneumonia, which may progress to acute respiratory distress syndrome [3,4].

Clinically, C-reactive protein (CRP) and procalcitonin serve as valuable biomarkers for inflammatory diseases [5,6]. Sepsis frequently co-occurs with underlying conditions such as autoimmune diseases, cancers, and advanced chronic liver disease [7,8]. Notably, patients with liver cirrhosis exhibit reduced CRP levels due to impaired hepatic synthesis [9], while procalcitonin levels in individuals with severe liver diseases tend to be elevated [10]. This highlights that circulating levels of these laboratory markers are influenced not only by inflammation but also by the underlying disease.

Adiponectin, a well-studied adipokine primarily produced in adipocytes, exhibits distinct gender-related variations, with higher circulating levels in females compared to males [11,12,13]. Importantly, reduced serum adiponectin levels are associated with obesity, contributing to metabolic dysregulation, including insulin resistance [11,12,14]. 

Beyond its crucial metabolic functions, adiponectin also possesses anti-inflammatory properties [11,12,13]. In studies involving lipopolysaccharide-exposed macrophages, adiponectin treatment led to decreased interleukin (IL)-6 levels and increased IL-10 expression [15]. Similarly, IL-10 levels were elevated in macrophages exposed to recombinant adiponectin [16]. In mice lacking adiponectin, peritoneal macrophages displayed a pro-inflammatory phenotype, suggesting an anti-inflammatory role for adiponectin [17]. Additionally, adiponectin was found to reduce the levels of CC-chemokine ligand-2 (CCL2) and C-X-C motif chemokine ligand 1 (CXCL1) induced by tumor necrosis factor or poly(I:C) in the human bronchial epithelium [14,18]. 

However, adiponectin was also shown to have pro-inflammatory effects; this protein activated nuclear factor kappa B (NF-kappaB) in different cell types, including macrophages, myocytes, and hepatocytes [19] and induced IL-6, CCL2, and CXCL8 expression in human monocytes [20]. Adiponectin forms trimers, hexamers, and high-molecular-weight forms, and these isoforms may exert different effects. It is also possible that the early inflammatory effects of adiponectin later on induce the synthesis of anti-inflammatory cytokines [19]. 

Circulating levels of adiponectin tend to be positively correlated with clinical markers of inflammation in various inflammatory diseases. Patients with heart failure, inflammatory bowel diseases, and rheumatoid arthritis had elevated plasma adiponectin in contrast to controls [19,21,22]. 

Studies that have determined serum adiponectin levels in patients with sepsis are comparably rare and have reported controversial results [23]. It was shown that critically ill patients had systemic adiponectin levels comparable to healthy controls [24]. Another study observed high serum adiponectin levels in sepsis patients in contrast to healthy controls. In the patient cohort, systemic adiponectin was not correlated with clinical markers of disease severity [25]. It was also reported that severely ill patients had low adiponectin levels, which normalized when the patients recovered [26,27]. Low plasma adiponectin in sepsis compared to pre-septic patients was described in a further study, and a modest increase in plasma adiponectin during sepsis onset was related to higher mortality [28]. An association of higher plasma adiponectin concentrations with mortality was also observed in patients with acute respiratory failure [29]. In a cohort of patients admitted to the intensive care unit, low adiponectin predicted overall survival [24]. 

Studies that analyzed the systemic adiponectin levels of patients with corona virus disease 2019 (COVID-19) also reported discordant results. Adiponectin was found to be reduced in the plasma of severely ill COVID-19 patients in contrast to controls and patients with mild disease. This analysis showed that plasma adiponectin was low in patients with severe illness, irrespective of the infectious agent, and thus indicated that reduced systemic adiponectin levels were not a cause of SARS-CoV-2 infection [30]. A separate study reported higher serum adiponectin levels in COVID-19 patients in comparison to healthy controls. In this cohort, adiponectin levels were not related to severe pneumonia or outcome [31]. Along these lines, most of the studies published so far could not identify associations between adiponectin levels and COVID-19 disease severity [32,33,34,35]. 

Females have higher adiponectin levels in comparison to males [11,12,13], but most clinical studies in severely ill patients did not account for sex differences. The aim of our study was a sex- and disease-specific analysis of plasma adiponectin levels in critically ill patients.

## 2. Materials and Methods

### 2.1. Study Cohort

Between August 2018 and January 2023, we collected plasma samples from 156 patients at the University Hospital of Regensburg. These patients had various causes of systemic inflammatory response syndrome (SIRS, 37 patients), sepsis (40 patients), or septic shock (79 patients). Plasma samples from 23 COVID-19 patients were obtained between October 2020 and January 2023. It is important to note that all COVID-19 patients included in our study were in sepsis or septic shock due to SARS-CoV-2 infections.

We categorized patients using the Sepsis-3 criteria for sepsis and septic shock [36] and the SIRS criteria for SIRS [37]. Patients with viral hepatitis, human immunodeficiency virus infection, or multi-resistant infections were excluded. Laboratory values were obtained from the Institute of Clinical Chemistry and Laboratory Medicine at the University Hospital Regensburg, while microbiological tests were conducted by the Institute of Clinical Microbiology and Hygiene at the same hospital.

### 2.2. Adiponectin and Interleukin-6 (IL-6) ELISAs

We collected blood samples from patients within 12 to 24 h of their admission to the intensive care unit using EDTA as the anticoagulant. Plasma was separated from the blood samples. We employed the human Adiponectin DuoSet ELISA kit (R&D Systems; Wiesbaden, Nordenstadt, Germany) following the manufacturer’s instructions, with a plasma dilution of 1:5000 for analysis. The human IL-6 DuoSet ELISA kit from R&D Systems was used for IL-6 analysis, and plasma was 2-fold diluted. 

### 2.3. Statistical Analysis

We represented the data using boxplots, which visually display the minimum and maximum adiponectin values, the median, and the first and third quartiles. Outliers are indicated as individual circles or asterisks. The tables provide details on the median, minimum, and maximum values. We applied statistical tests including the non-parametric Mann–Whitney-U-test, the non-parametric Kruskal–Wallis test, the Chi–Square-Test, multiple linear regression, the receiver operating characteristic curve, and Spearman’s correlation using IBM SPSS Statistics 26.0 software. A significance level of *p* < 0.05 was used.

The cohort size was estimated using data from previous studies that analyzed the adiponectin levels of sepsis patients and controls, and G*Power 3.1.6. According to the findings by Vassiladi et al. [25], 74 patients and 12 controls were needed (effect size d: 1.05, α = 0.05, 1 − β = 0.95). According to the study by Venkatesh et al. [27], 27 patients and 5 controls were necessary (effect size d: 1.79, α = 0.05, 1 − β = 0.95). This shows that our cohort was large enough to identify such differences in plasma adiponectin levels between patients and controls, even when patients with liver cirrhosis were excluded. 

## 3. Results

### 3.1. Adiponectin in the Plasma of Systemic Inflammatory Response Syndrome/Sepsis Patients with and without Liver Cirrhosis

Adiponectin was determined in the plasma of 156 patients with systemic inflammatory response syndrome (SIRS)/sepsis and 22 controls. Patients and controls were matched for sex and age (Table 1). Sepsis patients had higher plasma interleukin-6 (IL-6) levels in comparison to the controls (Table 1). A comparison of the whole study cohort and the subgroup of patients without liver cirrhosis showed that this latter group had higher levels of C-reactive protein (CRP). All other parameters listed in Table 1 were similar between the whole SIRS/sepsis cohort and the SIRS/sepsis patients without liver cirrhosis. 

**Figure 1:**
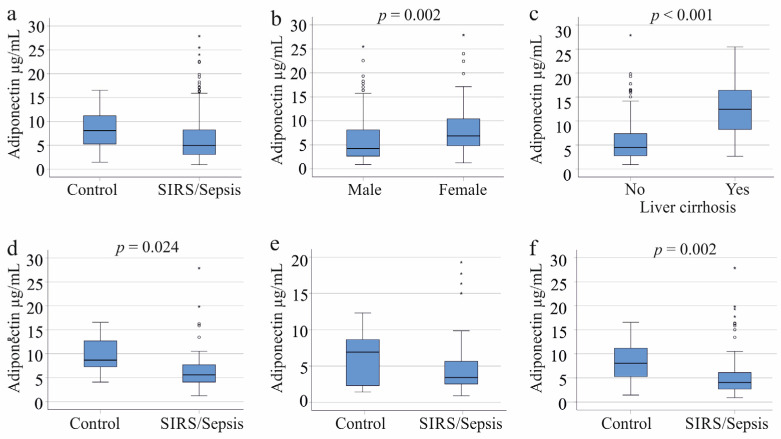
Adiponectin in the plasma of controls and systemic inflammatory response syndrome (SIRS)/sepsis patients. (**a**) Plasma adiponectin levels of the 22 controls and the 156 SIRS/sepsis patients; (**b**) Plasma adiponectin levels of the 109 male and 47 female SIRS/sepsis patients; (**c**) Plasma adiponectin levels of 32 SIRS/sepsis patients with and 124 SIRS/sepsis patients without liver cirrhosis; (**d**) Plasma adiponectin levels of 11 female controls and 38 female SIRS/sepsis patients when patients with liver cirrhosis were excluded; (**e**) Plasma adiponectin levels of 11 male controls and 86 male SIRS/sepsis patients when patients with liver cirrhosis were excluded; (**f**) Plasma adiponectin levels of 22 controls and 124 SIRS/sepsis patients of both sexes when patients with liver cirrhosis were excluded. Statistical test used: Mann–Whitney-U-test.

Among the 156 patients with SIRS or sepsis, the median plasma adiponectin concentration was 4.9 µg/mL, with a range from 0.9 to 27.9 µg/mL. In comparison, the 22 controls had a median concentration of 8.0 µg/mL, ranging from 1.4 to 16.5 µg/mL. Although lower in the patient’s plasma, the difference was not significant (*p* = 0.055; Figure 1a). Men had lower adiponectin levels than women (*p* = 0.002; Figure 1b). However, when comparing by gender, adiponectin levels between male patients and controls and between female patients and controls were not statistically different (*p*-values of 0.726 and 0.105, respectively).

In the entire cohort, plasma adiponectin did not show a significant correlation with age (correlation coefficient r = 0.106, *p* = 0.189) or body mass index (BMI) (correlation coefficient r = −0.046, *p* = 0.570). Among females, the correlation with age was also not significant (r = −0.188, *p* = 0.217). Plasma adiponectin in females was negatively correlated with BMI (correlation coefficient r = −0.432, *p* = 0.003). However, for males, a significant positive correlation was observed between plasma adiponectin levels and age (r = 0.190, *p* = 0.048), but not with BMI (correlation coefficient r = 0.102, *p* = 0.291).

There is consensus that patients with liver cirrhosis exhibit increased adiponectin levels [24,38]. Plasma adiponectin levels in cirrhosis patients were significantly higher compared to those without cirrhosis (*p* < 0.001, Figure 1c). Among male patients, 23 had cirrhosis, and they also showed a significant increase in adiponectin levels (*p* < 0.001). Similarly, among female patients, 9 had liver cirrhosis, and their adiponectin levels were significantly elevated (*p* = 0.005).

The proportion of patients with SIRS, sepsis, and septic shock was similar between the groups. Gender distribution, age, procalcitonin, IL-6, and leukocyte counts did not differ between these groups (Table 1). 

Because of the high plasma adiponectin levels of patients with cirrhosis, these patients were not included in the further analyses.

Female SIRS/sepsis patients had significantly lower plasma adiponectin levels than controls (*p* = 0.024). In contrast, male SIRS/sepsis patients did not show a significant difference from controls (*p* = 0.187). Overall, the adiponectin levels in the entire cohort of SIRS/sepsis patients were significantly reduced compared to controls (*p* = 0.002) (Figure 1d–f).

### 3.2. Plasma Adiponectin of SIRS/Sepsis Patients Stratified for SIRS, Sepsis, and Septic Shock and Underlying Diseases 

**Figure 2:**
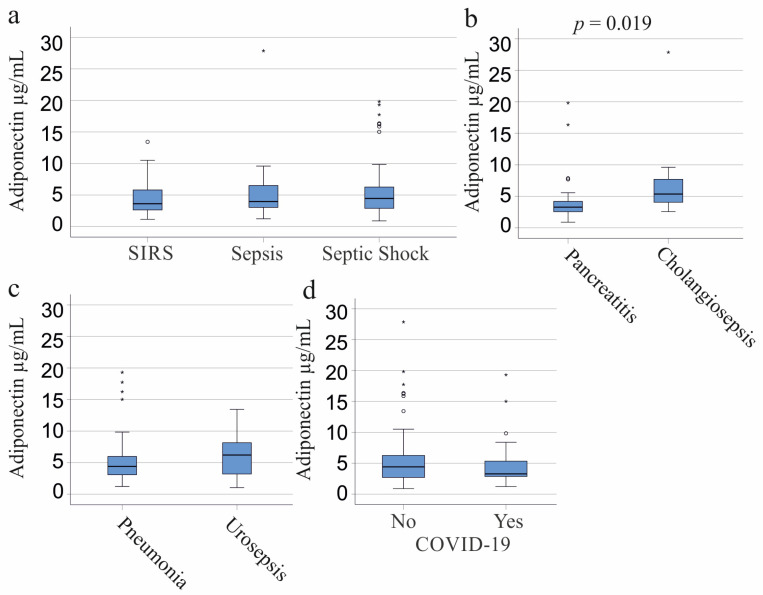
Adiponectin in the plasma of patients with systemic inflammatory response syndrome (SIRS)/sepsis stratified according to the underlying diseases and causes of SIRS/sepsis. (**a**) Plasma adiponectin of patients categorized according to the SIRS criteria and to the Sepsis-3 definition as SIRS (27 patients), sepsis (33 patients), and septic shock (64 patients); (**b**) Plasma adiponectin levels of SIRS/sepsis patients with pancreatitis (31 patients) or cholangiosepsis (9 patients); (**c**) Plasma adiponectin levels of SIRS/sepsis patients with pneumonia (41 patients) or urinary tract infections (14 patients); (**d**) Plasma adiponectin of 23 SIRS/sepsis patients with SARS-CoV-2 infection (Yes) and patients not infected by this virus (No). Statistical tests used: Kruskal–Wallis test (**a**), Mann–Whitney-U-test (**b**–**d**).

Patients were classified into three categories: SIRS, septic shock, and sepsis [37]. There was no significant difference in circulating adiponectin levels across these groups, whether viewed as a whole cohort (*p* = 0.832), males (*p* = 0.360), or females (*p* = 0.257) (Figure 2a). Of our cohort, 31 patients developed SIRS/sepsis from pancreatitis and 9 from cholangitis. Patients with pancreatitis exhibited lower plasma adiponectin levels compared to those with cholangiosepsis (*p* = 0.019, Figure 2b) and compared to all other patients without pancreatitis (*p* = 0.015). Both conditions were diagnosed in four women each, with no significant difference in adiponectin levels between them (*p* = 0.386) or between females with and without pancreatitis (*p* = 0.915). Among men, the 27 with pancreatitis and the 5 with cholangiosepsis had comparable adiponectin levels (*p* = 0.175). Male patients with pancreatitis tended to exhibit lower adiponectin levels than those without pancreatitis, though this trend was not significant (*p* = 0.062).

### 3.3. Plasma Adiponectin of SIRS/Sepsis Patients Stratified for Infectious Diseases, SARS-CoV-2, and Bacterial Infections

Common infections that lead to sepsis are pulmonary (41 patients) and urinary tract infections (14 patients). Plasma adiponectin levels were similar between these groups of patients (*p* = 0.297; Figure 2c). The 11 females with pneumonia tended to have lower adiponectin than the 9 urosepsis patients (*p* = 0.067). The 30 men with pneumonia and the 5 men with urosepsis had comparable plasma adiponectin levels (*p* = 0.202). 

SARS-CoV-2 infection is a more recent cause of sepsis [39,40]. Among the six female COVID-19 patients, plasma adiponectin was lower compared to non-infected females (*p* = 0.008). Age, BMI, IL-6, and leukocyte counts did not differ between females with and without SARS-CoV-2 infection. 

However, there were no significant differences in adiponectin levels between the 17 male COVID-19 patients and non-infected males (*p* = 0.657) or in the whole cohort (*p* = 0.367) (Figure 2d). CRP and leukocyte counts were similar between the groups, but COVID-19 patients exhibited lower procalcitonin levels (*p* = 0.027). This reduction in procalcitonin was significant in infected females (*p* = 0.008) but not in males (*p* = 0.657). Moreover, female COVID-19 patients displayed lower procalcitonin levels than their male counterparts (*p* = 0.006), while there was no significant difference between the procalcitonin levels of non-infected males and females (*p* = 0.830). Notably, SARS-CoV-2-infected males had a lower number of eosinophils (*p* = 0.013) in comparison to males without SARS-CoV-2 infection. Age, BMI, IL-6, CRP, and the number of other white blood cells did not differ between these two groups (*p* > 0.05 for all). 

### 3.4. Plasma Adiponectin Levels in Relation to Vasopressor Therapy and Interventions

The associations of plasma adiponectin levels with the need for dialysis, ventilation, or vasopressor treatment were calculated for the SIRS/sepsis patients who did not suffer from liver cirrhosis (Table 2). Plasma adiponectin levels were not related to any of these measures in the whole cohort or in the sex-specific analysis (Table 2). 

### 3.5. Plasma Adiponectin Levels in Relation to Inflammation Markers

In both the entire cohort and among male patients, a negative correlation was identified between plasma adiponectin and CRP. No correlations were detected with neutrophils, basophils, eosinophils, monocytes, lymphocytes, immature granulocytes, IL-6, or procalcitonin, either in the whole cohort or in sex-specific analyses (Table 3).

### 3.6. Plasma Adiponectin Levels in Gram-Negative and Gram-Positive Infection

**Figure 3:**
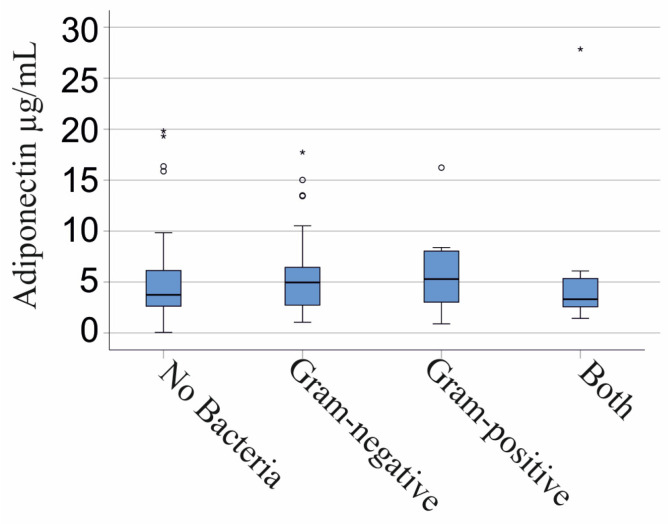
Adiponectin in the plasma of patients with SIRS/sepsis is stratified by type of bacterial infection. A total of 44 patients with no bacterial infections, 51 Gram-negative infected patients, 15 patients with Gram-positive bacteria, and 14 patients with Gram-negative and Gram-positive bacteria. Statistical test used: Kruskal–Wallis test.

Plasma adiponectin of the 44 patients with no bacterial infections was comparable with the levels of the 51 Gram-negative infected patients, the 15 patients with Gram-positive bacteria, and the 14 patients with Gram-negative and Gram-positive bacteria in their blood cultures (*p* = 0.643; Figure 3). This applied for males (*p* = 0.613) and females (*p* = 0.646). 

Plasma adiponectin levels did not differ between the 20 patients infected only with *Escherichia coli* (*p* = 0.690) and the 21 patients monoinfected with *Enterococcus faecalis* (*p* = 0.553) in comparison to non-infected patients. The 3 patients monoinfected with *Staphylococcus aureus* (*p* = 0.727) and the 12 patients monoinfected with *Staphylococcus epidermidis* (*p* = 0.369) had similar plasma adiponectin levels as SIRS/sepsis patients, where no pathogens were identified. 

### 3.7. Multiple Regression Analysis

To analyze whether the variables that showed associations with plasma adiponectin levels in our SIRS/sepsis patients (BMI, CRP, age, sex, and liver cirrhosis) could predict plasma adiponectin levels, multiple regression analysis was performed. In the whole patient cohort, the R^2^ for the overall model was 0.357 (the adjusted R^2^ was 0.330), indicative of high goodness-of-fit [41]. BMI, CRP, age, sex, and liver cirrhosis predicted plasma adiponectin with a *p* < 0.001 (F(6/146). Sex (*p* = 0.001), cirrhosis (*p* < 0.001), and CRP (*p* = 0.016) were the significant predictors of plasma adiponectin levels. 

In the SIRS/sepsis cohort (excluding patients with liver cirrhosis), BMI, pancreatitis, COVID-19, age, and sex predicted plasma adiponectin with *p* = 0.038 (F(5/117). The R^2^ for the overall model was 0.095 (the adjusted R^2^ was 0.056), indicative of low goodness-of-fit [41]. In this analysis, sex was the only significant predictor (*p* = 0.002) of plasma adiponectin levels. 

Multivariate analysis including COVID-19, pancreatitis, pneumonia, and BMI in females was not significant (*p* = 0.053; F(4/33)). The R^2^ for the model was 0.241 (adjusted to 0.149). BMI was the only significant predictor (*p* = 0.023) of plasma adiponectin levels in females. 

Multivariate analysis was also performed to evaluate the impact of age and CRP on plasma adiponectin levels in males, and this was significant (*p* = 0.015; F(2/83)). The R^2^ for the model was 0.096 (adjusted 0.074), indicative of a modest goodness-of-fit [41]. CRP was the most significant predictor (*p* = 0.035) of plasma adiponectin levels in males. 

### 3.8. Plasma Adiponectin Levels and Survival

**Figure 4:**
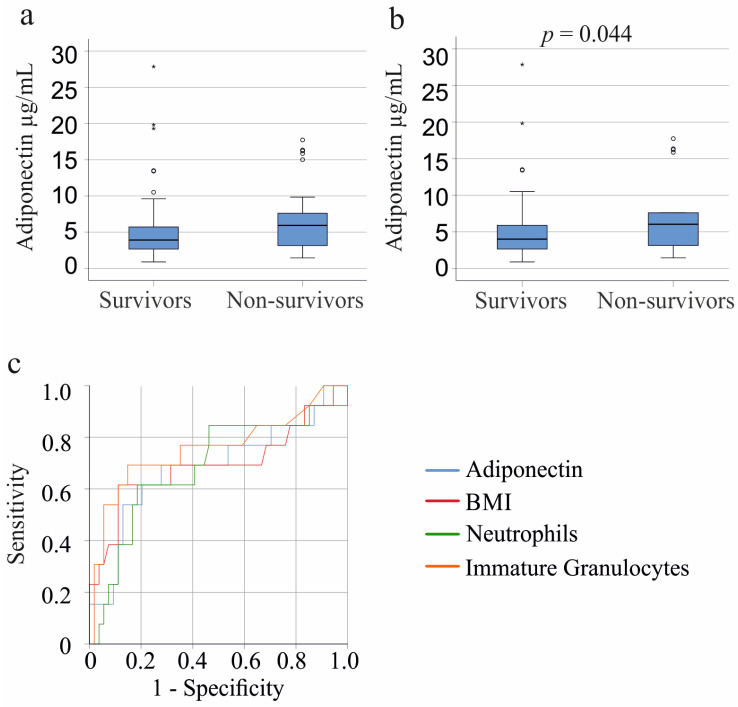
Association of plasma adiponectin with survival. (**a**) Plasma adiponectin levels of the 99 systemic inflammatory response syndrome (SIRS)/sepsis patients who survived and the 25 patients who did not survive; (**b**) Plasma adiponectin levels of the 84 SIRS/sepsis patients who survived and the 18 patients who did not survive when patients with COVID-19 and liver cirrhosis were excluded. Statistical test used: Mann–Whitney-U-test; (**c**) Receiver operating characteristic curve for non-survival of male SIRS/sepsis patients (13 patients died and 57 survived) with COVID-19 and liver cirrhosis were excluded.

Plasma adiponectin of the 25 patients (patients with liver cirrhosis were excluded) who did not survive showed a trend to be higher in contrast to survivors (*p* = 0.074; Figure 4a). This tendency was observed for the 19 male non-survivors (*p* = 0.064) but not for the 6 female non-survivors (*p* = 0.399). The male patients who did not survive had a higher BMI (*p* = 0.041) and more immature granulocytes (*p* = 0.030). Such differences were not significant in females. 

Of the COVID-19 cohort, 7 patients died; however, their adiponectin levels were similar to those who survived (*p* = 0.799). Among patients with liver cirrhosis (excluding those with COVID-19), 11 died, and their plasma adiponectin levels were comparable to survivors (*p* = 0.967). When excluding both liver cirrhosis and COVID-19 patients, the 18 non-survivors showed significantly higher plasma adiponectin levels (*p* = 0.044) (Figure 4b). Age, BMI, CRP, and IL-6 did not differ between survivors and non-survivors (*p* > 0.05). Non-survivors had more neutrophils (*p* = 0.005) and immature granulocytes (*p* = 0.002). 

Male survivors had higher adiponectin levels (*p* = 0.036), more neutrophils (*p* = 0.039), immature granulocytes (*p* = 0.004), and a higher BMI (*p* = 0.032) than the male non-survivors. 

Receiver operating curve analysis for non-survival of males showed an area under the curve of 0.686 for neutrophils, 0.757 for immature granulocytes, 0.694 for BMI, and 0.687 for adiponectin. Multivariate analysis including BMI, neutrophil count, immature granulocytes, and adiponectin significantly predicted death (*p* < 0.001, R^2^ = 0.331; F(4/62) in male patients (liver cirrhosis and COVID-19 patients were excluded). Immature granulocytes (*p* = 0.008) and BMI (*p* = 0.004) were the significant predictors of non-survival; the *p*-value for adiponectin was 0.060 and for neutrophils 0.769. 

Conversely, adiponectin levels, white blood cells, and inflammatory proteins, as well as age and BMI, in the 5 female non-survivors were similar to those in female survivors (*p* = 0.361).

## 4. Discussion

This study revealed that female patients with SIRS/sepsis have lower plasma adiponectin levels compared to healthy controls once patients with liver cirrhosis are excluded. Notably, liver cirrhosis patients, irrespective of gender, exhibited markedly elevated plasma adiponectin levels. Moreover, there was no observed association between adiponectin levels and disease severity, though levels tended to be higher in non-survivors.

Liver cirrhosis increases the risk of sepsis and mortality [7]. Septic patients with liver cirrhosis exhibited approximately 3-fold higher plasma adiponectin levels compared to SIRS/sepsis patients without cirrhosis. This pattern was consistent across both sexes. These findings highlight the potential of adiponectin as a marker for liver cirrhosis among SIRS/sepsis patients.

Pancreatitis, an inflammatory disease that can progress to sepsis [42], was associated with lower plasma adiponectin levels across our cohort. Given that adiponectin is known to reduce insulin resistance [43], its diminished levels in septic pancreatitis patients might help predict post-pancreatitis diabetes. For male patients presenting with unexplained SIRS/sepsis, low adiponectin levels might hint at an underlying pancreatitis. Moreover, in septic patients from other causes, reduced adiponectin could signify pancreatic complications, including secondary pancreatitis from interventions like endoscopy or surgery or even due to carcinoma. Future research is needed to validate these hypotheses.

The prevalence of underlying diseases (e.g., liver cirrhosis) and inflammation causes (e.g., pancreatitis, cholangitis, pneumonia, and urosepsis) in our cohort showed no significant gender differences. However, examining data from all German hospital admissions between 2005 and 2018 reveals that 64.8% of cirrhosis patients were male [44]. In our SIRS/sepsis group, liver cirrhosis appeared in 21% of males and 15% of females. Chronic pancreatitis, more common in males [45], was diagnosed in 26% of our male and 9% of our female subjects. Females have a higher risk for cholangitis [46], and 9% of female and 5% of male SIRS/sepsis patients had cholangiosepsis in our study. Urosepsis, whose risk is not gender-related [47], appeared in 19% of our female and 5% of our male patients. Pneumonia was present in 23% of females and 28% of males, with most studies indicating males have a higher community-acquired pneumonia incidence [48]. More males typically experience sepsis [49], as reflected in our male-predominant cohort. Overall, our sample size was adequate to identify gender prevalence in sepsis, while subgroup analysis failed, most likely because the resulting subgroups were too small.

Evidence suggests that the male gender is a risk factor for severe COVID-19 [3]. In our cohort, the representation of COVID-19 patients was balanced between genders, with 13% of females and 14% of males. Interestingly, the six female patients infected with SARS-CoV-2 displayed reduced plasma adiponectin levels when compared to non-infected women, a phenomenon not observed in the male cohort. 

Eosinopenia in sepsis is related to a poor outcome [50] and confers an increased risk for severe disease in COVID-19 [51]. Male COVID-19 patients had fewer eosinophils in comparison to male patients not infected by this virus, and such a difference was not observed in females. Data about variations in eosinophil counts between sepsis patients with and without SARS-CoV-2 infection are inconsistent [52,53]. Our data indicate a male-specific decline of eosinophils in COVID-19 patients, which is independent from disease severity. 

Due to the limited number of female COVID-19 patients in our study, further investigation in larger cohorts is essential to validate these sex-specific observations. While most studies to date have found no associations between adiponectin levels and COVID-19 infection or disease severity [32,33,34,35], which aligns with our findings in the broader cohort and among male patients, a distinct sex-specific analysis on this topic, to our knowledge, has yet to be conducted.

BMI negatively correlates with circulating adiponectin in the normal population [11,54]. In accordance with the study by Nooijer et al., plasma adiponectin did not correlate with BMI in our SIRS/sepsis cohort [55], and this might indicate that body weight-related changes in plasma adiponectin disappear in sepsis. Sex-specific analysis identified a negative association between plasma adiponectin and BMI in females, which was not observed in males. Multivariate analysis showed that BMI is the most significant predictor of plasma adiponectin levels in female SIRS/sepsis patients.

Obesity is a risk factor for severe COVID-19 but was also associated with a lower mortality rate in patients with sepsis [3,56]. In our cohort, a higher BMI was related to mortality in males, and a nearly significant effect of increased adiponectin levels on mortality was also noticed. This indicates complex interactions between sepsis severity, survival, adiponectin levels, and obesity. 

Studies that analyzed the serum adiponectin levels of patients with sepsis reported controversial results [23]. Critically ill patients had systemic adiponectin levels comparable to those of healthy controls [24], but high as well as low serum adiponectin levels in contrast to healthy controls were also observed [25,26,27]. The present analysis showed that the plasma adiponectin of SIRS/sepsis patients and controls is similar when patients with liver cirrhosis are included. Female SIRS/sepsis patients without cirrhosis had low plasma adiponectin in comparison to female controls, and such a difference did not occur in males. This suggests that underlying liver cirrhosis and sex may in part contribute to the discordant findings published so far [23,25,26,27,28]. 

In agreement with previous observations [25], plasma adiponectin was not related to disease severity. Female and male patients requiring dialysis, ventilation, or vasopressor therapy had similar plasma adiponectin levels as patients with no need for these interventions. 

Bacterial infection in male and female SIRS/sepsis patients was not related to a change in plasma adiponectin levels. Patients infected with *Escherichia coli*, *Enterococcus faecalis*, *Staphylococcus aureus*, or *Staphylococcus epidermidis* had similar plasma adiponectin levels as SIRS/sepsis patients, where no pathogen was identified. 

The major pathogens for urinary tract infections are *E. coli* and *Klebsiella pneumoniae*, and for pneumonia, the most common pathogen is *Streptococcus pneumoniae* [57]. Plasma adiponectin levels of our patients with urosepsis or respiratory infections were similar, further indicating that the type of pathogen has no effect on plasma adiponectin levels. The bacterial cell surface contains lipopolysaccharide, which was found to downregulate adiponectin in adipose tissue. Peptidoglycan is highly abundant in Gram-positive bacteria, and this compound increased adiponectin levels in adipose tissues [58]. These ex vivo findings do not translate to altered levels of plasma adiponectin in SIRS/sepsis patients infected with different bacteria. 

Plasma adiponectin levels in both sexes did not correlate with procalcitonin, neutrophils, basophils, eosinophils, monocytes, lymphocytes, or immature granulocytes. Patients with sepsis are commonly characterized by neutrophilia, lymphocytopenia, and eosinopenia [59], and from the present results, these features are not related to adiponectin. There was a modest negative correlation between plasma adiponectin and CRP in male but not female patients. Multiple linear regression analyses revealed CRP to significantly predict plasma adiponectin levels in males. This might indicate that adiponectin still exerts some anti-inflammatory activity in males. However, observational studies can only describe associations and cannot provide experimental proof for this assumption. 

Plasma adiponectin levels were found to be induced in non-survivors, and similar observations have been published before. In this previous study, a modest increase in plasma adiponectin in early sepsis was related to higher mortality [28]. Notably, associations between adiponectin and survival were not found in another cohort of sepsis patients [35]. It was also reported that low adiponectin is related to mortality in sepsis patients [60]. Current analysis suggests that associations of plasma adiponectin with survival are modified by sex, underlying liver cirrhosis, and SARS-CoV-2 infection. Multivariate analysis including BMI, neutrophil count, immature granulocytes, and adiponectin significantly predicted death in male patients when patients with liver cirrhosis and COVID-19 patients were excluded. The clinical value of different white blood cell counts for predicting mortality in sepsis patients has not been finally clarified [61,62], and sex-related analysis may be needed. Most cohorts, including ours, are too small to account for all of the confounding factors, and the relationship between adiponectin and survival in SIRS/sepsis patients needs further study. 

This study has limitations. Sex-specific analysis of female subgroups was limited by the low number of patients, and confirmatory experiments are required. BMI is well described to negatively correlate with plasma adiponectin in the normal population [11,54] but was not documented for the control cohort in our study. However, all of our controls were normal-weight. 

## 5. Conclusions

Awareness of the influence of sex on the pathophysiology of various diseases is growing [63]. Our study highlights sex-specific differences and similarities in plasma adiponectin levels among SIRS/sepsis patients. Clinically, these levels were also linked to underlying conditions like liver cirrhosis and pancreatitis. Elevated adiponectin levels might indicate liver cirrhosis in SIRS/sepsis patients, while decreased levels could suggest pancreatitis, potentially predicting post-pancreatitis diabetes. Therefore, associations between plasma adiponectin levels, critical illness, and mortality seem to be influenced by BMI, sex, and disease etiology. However, our findings necessitate validation through larger, multicentric studies.

## Figures and Tables

**Table 1:** Characteristics of the controls, the systemic inflammatory response syndrome(SIRS)/sepsis patients, and the SIRS/sepsis patients where patients with liver cirrhosis were excluded. Interleukin (IL)-6 was not determined in all patients because of the lack of plasma, and the number of patients where IL-6 could be measured is given in upper case. The controls were healthy, and all had a normal body weight. The statistical tests used were the Mann–Whitney-U-test and the Chi–Square-Test. *** *p* < 0.001 for comparison of patients with and without liver cirrhosis, ^%%%^ *p* < 0.001 for comparison of patients and controls.

Parameters	All Patients	Patients with Liver Cirrhosis Excluded	Controls
Males/Females	109/47	86/38	11/11
Age (years)	59 (21–93)	57 (21–88)	58 (40–67)
Body Mass Index (kg/m^2^)	26.2 (15.4–55.6)	26.2 (15.4–55.6)	not defined
SIRS/Sepsis/Septic Shock	37/40/79	27/33/64	not defined
C-reactive protein mg/L	157 (12–697) ***	183 (35–597) ***	not defined
Procalcitonin ng/mL	1.15 (0.05–270.00)	1.17 (0.06–270.00)	not defined
IL-6 pg/mL	89 (0–5702)^150 %%%^	75 (0–5702)^121 %%%^	7 (0–48)^21 %%%^
Leukocytes n/nL	10.31 (0.06–1586.00)	10.35 (2.16–37.38)	not defined
Neutrophils n/nL	7.55 (0–70.20)	7.34 (0–70.20)	not defined
Basophils n/nL	0.04 (0–0.90)	0.04 (0–0.90)	not defined
Eosinophils n/nL	0.13 (0–8.80)	0.12 (0–8.80)	not defined
Monocytes n/nL	0.78 (0–45.00)	0.75 (0–45.00)	not defined
Lymphocytes n/nL	0.95 (0.08–28.60)	1.04 (0.08–28.60)	not defined
Immature Granulocytes n/nL	0.12 (0–6.19)	0.12 (0–6.19)	not defined

**Table 2:** Comparison of plasma adiponectin levels between patients with dialysis, ventilation, and vasopressor therapy and patients without these conditions. The number of patients treated is given in “N,” and the respective *p*-values are listed. Statistical test used: Mann–Whitney-U-test.

Intervention/Drug	Patients without Liver Cirrhosis (124)		Females without Liver Cirrhosis (38)		Males without Liver Cirrhosis (86)	
	N	*p*-Value	N	*p*-Value	N	*p*-Value
Dialysis	38	0.160	9	0.325	29	0.185
Ventilation	75	0.994	23	0.137	52	0.500
Vasopressor therapy	74	0.530	19	0.298	55	0.140

**Table 3:** Correlation coefficient (r) and *p*-values for plasma adiponectin and its associations with clinical markers of inflammation. Statistical test used: Spearman correlation.

Biomarker of Inflammation	Patients without Liver Cirrhosis (124)		Females without Liver Cirrhosis (38)		Males without Liver Cirrhosis (86)	
	r	*p*-Value	r	*p*-Value	r	*p*-Value
Leukocytes	−0.028	0.759	−0.085	0.612	0.055	0.616
Neutrophils	−0.003	0.977	−0.114	0.502	0.149	0.177
Basophils	0.020	0.825	0.227	0.171	−0.001	0.994
Eosinophils	−0.131	0.151	0.236	0.154	−0.195	0.077
Monocytes	−0.023	0.804	−0.171	0.306	0.114	0.304
Lymphocytes	−0.114	0.215	0.041	0.805	−0.142	0.202
Immature Granulocytes	−0.083	0.372	−0.197	0.250	0.022	0.846
Procalcitonin	−0.020	0.825	0.310	0.058	−0.141	0.204
C-reactive protein	−0.199	0.027	−0.067	0.688	−0.299	0.005
Interleukin-6	−0.022	0.807	0.159	0.341	−0.098	0.379

## Data Availability

Data supporting reported results can be obtained from the corresponding author.
